# Challenges and Advances in the Production of Transplantable Retinal Tissue from Retinal Organoids

**DOI:** 10.18502/jovr.v20.17499

**Published:** 2025-06-18

**Authors:** Samir Malhotra, Magdalene J. Seiler, Andrew W. Browne

**Affiliations:** ^1^Department of Biomedical Engineering, University of California, Irvine, Irvine, CA, USA; ^2^Sue & Bill Gross Stem Cell Research Center, University of California, Irvine, Irvine, CA, USA; ^3^Center for Translational Vision Research, University of California, Irvine, Irvine, CA, USA; ^4^Department of Physical Medicine & Rehabilitation, School of Medicine, University of California, Irvine, Irvine, CA, USA; ^5^Department of Anatomy & Neurobiology, School of Medicine, University of California, Irvine, Irvine, CA, USA; ^6^Department of Ophthalmology & Visual Sciences,, Gavin Herbert Eye Institute, University of California, Irvine, Irvine, CA, USA

**Keywords:** Photoreceptors, Pluripotent Stem Cells, Regenerative Medicine, Retinal Degeneration, Retinal Organoids, Retinal Transplantation

## Abstract

Retinal degenerative diseases (RDD), which impair photoreceptors, the retinal pigment epithelium (RPE), and associated retinal cells, result in severe vision loss. For patients with advanced RDD, tissue replacement therapies, such as transplantation, offer potential pathways to visual rehabilitation. While fetal retinal transplantation has shown some promise in preclinical and clinical studies, human pluripotent stem cell (hPSC)-derived retinal organoids (ROs) present a promising alternative. ROs are three-dimensional tissues that replicate key aspects of retinal development, making them viable candidates for transplantation. However, the path toward clinical application faces two primary challenges: achieving Good Manufacturing Practice (GMP)-compliant production and overcoming technical difficulties associated with safe transplantation. Current RO production protocols are often limited by variability in tissue morphology, yield, and reproducibility, while transplantation efforts are hindered by rosette formation and mechanical damage to the subretinal space. Recent innovations, including automated bioreactor systems and optimized surgical techniques, offer potential solutions. Further advances in understanding and preventing rosette formation are essential to improve transplantation outcomes. Continued research and technological development are necessary to unlock the full potential of ROs for visual rehabilitation in patients with retinal degeneration.

##  INTRODUCTION

Retinal degenerative diseases (RDD) affect photoreceptors (PRs), the underlying retinal pigment epithelium (RPE) and surrounding retinal cells, leading to vision loss. For patients with advanced RDD, tissue replacement therapy, such as transplantation, offers hope for visual rehabilitation.

Transplantation of fetal retina in humans^[1]^ and preclinical models^[2,3,4]^ has demonstrated variable degrees of visual rehabilitation in severe stage disease. However, the use of fetal tissues introduces complexity and ethical challenges, spurring interest in alternative retinal tissue sources such as genetically modified organoids and other stem cell-derived retinal tissues. Compared to fetal retinal transplantation, these approaches are generally associated with fewer ethical constraints, offering a more broadly acceptable pathway for therapeutic development. Nevertheless, ethical considerations surrounding genetically modified organoids are important and merit careful examination in dedicated discussions.

Genetic therapies, such as gene augmentation and gene editing, hold promise for early-stage RDDs by targeting the molecular mechanisms that drive disease progression and seeking to prevent or halt further deterioration. However, these therapies are limited in their applicability to advanced stages of retinal degeneration, where the irreversible loss of PRs and supporting retinal tissues precludes their effectiveness. In such cases, cell replacement therapies, including manufactured cell sheets, dissociated cells, and retinal organoids (ROs), offer a viable alternative by directly addressing the need for structural and functional tissue restoration.^[5]^ Among these, ROs provide unique advantages, as their three-dimensional (3D) architecture recapitulates retinal development, overcoming challenges associated with integration and stratification in other approaches.

Human pluripotent stem cell (hPSC)-derived ROs have emerged as promising tools in regenerative medicine, offering structurally and physiologically relevant models for studying retinal development and disease. ROs are 3D tissues that recapitulate the cellular composition and developmental events of retinogenesis, making them suitable candidates for transplantation in vision restoration. Preclinical studies have demonstrated some efficacy in vision improvement after transplanting retinal sheets obtained from ROs.^[6,7,8]^ In this perspective, we discuss two significant impediments to reaching the goal of transplanting RO sheets for visual rehabilitation in humans.

### Challenges in Manufacturing ROs

**Figure 1:**
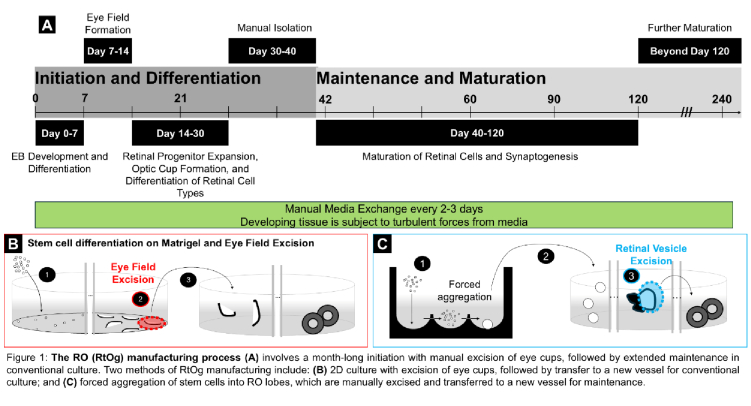
The retinal organoid manufacturing process (A) involves a month-long initiation with manual excision of eye cups, followed by extended maintenance in conventional tissue culture. Two methods of RtOg manufacturing include: (B) 2D culture with excision of eye cups, followed by transfer to a new vessel for conventional culture, and (C) forced aggregation of stem cells into retinal vesicles, which are manually excised and transferred to a new vessel for maintenance.

Sasai's group generated the first fully defined 3D ROs from mouse embryonic stem cells (mESCs),^[9]^ and later from hPSCs.^[10]^ The production of ROs generally follows a sequence [Figure 1A], where hPSCs are first encouraged, by one of two approaches, to assemble into one of two early retinal structures under chemically defined culture conditions^[11]^ that are then manually dissected. The first approach [Figure 1B] seeds stem cells onto Matrigel and supplies appropriate culture media while eye fields develop in a two-dimensional (2D) culture. These eye fields are visually identified, manually excised, and transferred into a different tissue culture vessel, where they continue developing into ROs. The second approach [Figure 1C] seeds stem cells into round-bottom wells, allowing them to aggregate. These aggregates are then transferred to a new tissue culture vessel, where 3D eye cup structures develop as lobes that bud from the larger structure. The organoid lobes are manually excised and maintained in conventional tissue culture while ROs develop. Despite efforts to manipulate tissue culture, media composition, and growth factors to enhance the homogeneity and yield of ROs, we and others have found it challenging to replicate these methods reliably,^[12,13]^ mainly because they require many and nuanced manual steps. Further, the maturation of ROs with fully functional PRs is a gradual process, with the formation of PRs with outer segments beginning around Day 90 and continuing to mature over time. Our group has observed the development of a robust outer segment layer by approximately Day 150 following PSC differentiation.^[14,15,16,17]^

Established protocols supplement media with exogenous factors at specific stages of retinal development to regulate signaling pathways and enhance retinal cell specification. RO differentiation from pluripotent stem cells is guided by precise manipulation of the culture environment using specific growth factors, small molecules, and inhibitors that regulate signaling pathways like bone morphogenetic protein (BMP), Wnt, Nodal, and transforming growth factor-beta (TGF- β ), which are crucial for retinal fate specification and maturation. For example, early Wnt inhibition and BMP4 activation promote eye field formation, while later additions of factors like retinoic acid support photoreceptor differentiation.^[13,17]^ By carefully timing these modifications, scientists mimic *in vivo* developmental cues, enabling the cells to self-organize into structured ROs. Despite advancements in current RO manufacturing, methods often face challenges, such as tissues with multilobular organoid morphology, which reduce the size of retinal sheets that could be used for transplantation, and variable reproducibility and production yield.^[18]^ These challenges likely arise from factors such as differences in stem cell lines used to produce ROs, variability in molecular factors used in manufacturing protocols, and manual mechanical manipulations that cause minor trauma to developing neurological tissues that are not experienced *in utero*.

Recent innovations have defined some biochemical manipulations in RO manufacturing to improve yield and homogeneity. Harkin et al achieved 100% efficiency in generating ROs of consistent size and shape across multiple hPSC lines by temporally activating BMP signaling pathway.^[19]^ Specifically, cellular aggregates were treated with BMP4 on Day 6, followed by a half-media exchange every three days until Day 16. BMP4 treatment resulted in a 2–2.5 fold increase in RO differentiation, as quantified by percent SIX6:GFP positive organoids seen with fluorescence microscopy, compared to traditional methods without BMP4 treatment. Isla-Magrané et al demonstrated that triiodothyronine (T3) and low all-trans retinoic acid (ATRA) levels promoted photoreceptor maturation in ROs.^[20]^ Additionally, Kelley et al observed accelerated rod photoreceptor differentiation, as indicated by rhodopsin expression on Day 120, when ROs were supplemented with 9-cis retinal but not with ATRA.^[21]^ In addition to high-yield homogenous RO manufacturing, an additional need exists for standardized Good Manufacturing Practice (GMP) to manufacture clinical-grade, transplantable tissues.^[22]^

GMP-compliant protocols for ROs must ensure xeno-free conditions and minimize hands-on labor. During optic cup development and continued differentiation, Matrigel and fetal bovine serum (FBS) are commonly used as supportive substrates along with key growth factors to promote RO growth.^[22]^ Additionally, the dissection and isolation of optic cups from surrounding non-optic tissue presents another challenge for achieving GMP compliance due to its labor-intensive and time-consuming nature. Regent et al have addressed this dissection issue by using a cell scraper for harvesting eye fields, while sustaining a 2.5 to 4.6-fold increase in RO production.^[23]^ We believe that Harkin et al have introduced a method that will accelerate RO production to GMP standards by minimizing human manipulation steps.^[19]^

In the final stage of RO development, regular media exchanges are required in multiple individual culture dishes. The development of ROs is a slow and labor-intensive process, particularly due to the need for manual media exchanges every two to three days. This requirement increases the workload, but automated robotic media exchange systems offer a potential solution to streamline the process. Furthermore, repeated opening and closing of tissue culture chambers pose a risk for contamination, a problem that can be mitigated by employing closed robotic systems. In addition, the mechanical turbulence caused by moving tissue culture plates could introduce unnecessary stress to the developing tissue that does not occur *in vivo*. While not all research groups have access to advanced robotic systems, a structurally programmed closed perfusion system represents a cost-effective alternative for labs aiming to produce GMP-grade ROs for applications, including transplantation. One approach to automated maintenance is autonomous bioreactors.^[22,24]^ Developing affordable bioreactor tools that integrate the Harkin protocol could accelerate GMP-style manufacturing for vision scientists hoping to generate organoids for their variety of uses.^[25]^

### Challenges in Transplanting ROs

Once a GMP-compliant RO protocol is established, the primary challenge becomes ensuring safe and effective transplantation for clinical applications. Successful transplantation requires the precise and controlled placement of ROs in the sub-retinal space to maintain tissue integrity and avoid damage. Rosette formation, which compromises the structural organization and function of the transplanted tissue, is a key concern during this process.

RO publications focus on standardizing manufacturing methods to generate optimal ROs for successful transplantation in *in vivo* animal models. While stem cell-derived ROs have shown improvements in visual function, improper functional integration into the host retina can occur due to rosettes. Watari et al developed a rigorous quality control method that identifies and removes unwanted cell types (off-target tissues) from ROs, reducing rosettes and potentially improving transplantation outcomes.^[26]^ iPSC-derived retinal sheets showed no tumor or adverse effects in tumorigenicity studies and demonstrated promising engraftment and maturation in the severely degenerated conditions during preclinical efficacy studies.

Another critical strategy to bring transplantable RO tissues to patients must address immunogenicity in preclinical models. While immunodeficient animal models have demonstrated the regenerative capacity of RO transplantation, translation to human applications requires understanding and mitigating immune responses in immunocompetent models. This includes the development of immunosuppressive regimens and strategies for inducing immune tolerance to ensure long-term graft survival and functionality. Incorporating such studies will bridge critical gaps in the translational pathway, ensuring ROs can effectively address both the disease context and immune challenges in clinical settings. We observed that immunosuppressive drugs do not adversely affect RO development and metabolism *in vitro*, and ROs exhibit low alloreactivity in mixed lymphocyte reactions. We have also observed no rejection of RO sheets transplanted into immunocompetent retinal degeneration models treated with immunosuppression.

Aramant and Seiler developed a pioneering method for fetal retinal tissue transplantation using a patented instrument that gently places donor tissue into the subretinal space,^[2,3]^ minimizing the risk of increased intraocular pressure from excess fluid. Although their method demonstrated the potential for morphological repair of damaged retinal areas, a significant number of transplants still resulted in rosette formation, which can be attributed to damage to the Bruch's membrane, host RPE, or retinal tissue. Improper positioning of the transplant within the vitreous also contributed to this complication. Avoiding such damage depends on the precise insertion of tissue at the correct angle, along with continuous advancements in instruments that enhance control and accuracy over time.

Clinical experience indicates that visual outcomes following subretinal transplantation are influenced by several factors related to the tissue microenvironment and the potential for traumatic forces applied to PRs and the RPE. Subretinal surgery trials have demonstrated limited or no visual improvement, with some cases resulting in worsened visual outcomes,^[27]^ but this highlights the critical sensitivity in manipulating tissues in the subretinal space. Reduced visual function associated with subretinal manipulations is believed to be due to mechanical damage to PRs and the RPE. Indeed, several groups have shown the safety and stability of transplanted monolayer sheets of RPE with minor complications only attributed to mechanical injury during sheet placement during surgery.^[28,29]^

Further, the duration of retinal detachment is correlated with reduced visual outcomes, as photoreceptor apoptosis is triggered by detachment, leading to irreversible damage.^[30]^ Apoptosis in detached PRs may result from abnormal microphysiological osmotic imbalances when PRs lose contact with the RPE, compounded by mechanical stress from fluid turbulence. This clinical observation in retinal detachment suggests that optimal co-culture of ROs with sheets of RPE, similar to those transplanted previously, may better protect PRs before and during transplantation to optimize visual rehabilitation.^[31]^

Minimizing trauma during tissue delivery is critical for successful transplantation. Current methods, such as inserting retinal sheets through sclerotomies and retinotomies, may not provide the minimally traumatic environment needed to preserve the normal architecture and polarity of PRs in the subretinal space. Manual manipulation and positioning of RO sheets pose additional challenges, and alternative techniques that reduce mechanical strain during insertion are required.

A noteworthy case in Japan involved the transplantation of RO sheets into human subjects with advanced retinitis pigmentosa. In this procedure, sheets of retinal tissue were manually isolated from organoids, loaded into a 24-gauge plastic cannula, and delivered into the subretinal space.^[27]^ However, high-resolution OCT imaging two years post-transplantation revealed the formation of photoreceptor rosettes,^[32]^ a phenomenon commonly observed in both preclinical models and human trials. This suggests that the current combination of tissue selection, mechanical dissection, and delivery methods is insufficient to prevent rosette formation, which disrupts the intended organization of the photoreceptor layer.

Efforts to minimize rosette formation in transplanted tissues should focus on several key areas: (1) determining the extent to which *in vitro* manipulation of ROs correlates with rosette formation, (2) identifying organoids free of rosettes prior to transplantation, and (3) refining mechanical techniques to avoid damaging retinal tissues during the preparation and delivery of organoid sheets. Addressing these challenges will be critical to improving the efficacy and safety of RO transplantation for visual improvement.

Given the complexities of avoiding rosette formation and ensuring the integrity and functionality of transplanted tissue, validating ROs prior to transplantation is essential. Watari et al developed a quality control strategy to optimize the selection of retinal tissue, increasing the likelihood of successful transplantations.^[26]^ This technique involves dissecting the organoid into two distinct regions: the inner-central sheet or “cap,” derived from the polar regions of the organoid, and the outer-peripheral sheet or “ring,” originating from the equatorial region. The cap, which contains well-differentiated retinal tissue, is designated for transplantation, while the ring—more prone to off-target tissues—undergoes qPCR-based testing to ensure suitability. This approach ensures that only the most functionally appropriate retinal tissue from the organoid poles is transplanted, with equatorial tissues screened to confirm the absence of off-target differentiation.

While validation of ROs to confirm the presence of correct retinal cells improves the chances of successful transplantation, ensuring optimal synaptic connectivity is the key to confirming graft success. Synaptic connectivity is the functional linking of neurons through synapses, and repairing these connections between the graft and host retinal network is essential for restoring vision. Matsuyama et al and Yamasaki et al used genetic modifications to reduce secondary retinal cells, such as bipolar cells, either by knocking out Islet-1 (ISL1) or Bhlhb4 in stem cell-derived retinal grafts.^[33,34]^ This reduction of bipolar cells enhanced synaptic integration between the grafted PRs and host bipolar cells, leading to significant restoration of light responses and visual function. Additionally, the knockout grafts exhibited reduced spontaneous activity in degenerated retinas, resulting in better visual recovery. Transplantable tissues produced with RO technology and with optimal engraftment potential, whether derived from autologous or allogenic, may benefit from pharmacological or genetic modifications that limit bipolar cell development in ROs.

##  CONCLUSION

ROs derived from hPSCs present a promising avenue for addressing vision loss in patients with advanced RDD. Despite their potential, significant challenges remain in the manufacturing and transplantation of ROs. Ensuring GMP-compliant protocols for RO production requires addressing issues of tissue variability, labor-intensive processes, and the need for xeno-free conditions. Biochemical advancements and the use of automated bioreactors have shown promise in improving the yield and homogeneity of ROs, which is critical for clinical applications. However, the successful transplantation of RO sheets remains hindered by rosette formation and the potential for mechanical damage during the procedure. Refining tissue delivery methods to minimize trauma, improving *in vitro* handling techniques, and co-culturing ROs with RPE sheets are essential to optimize transplantation outcomes.

In addition to overcoming these technical challenges, combining gene therapy and cell replacement therapy could offer a comprehensive and synergistic approach to treating RDD. While cell replacement therapies, such as ROs or cell sheets, directly address tissue loss, gene therapy could be employed simultaneously to halt further disease progression and preserve remaining native retinal tissue. Furthermore, gene editing technologies offer the potential to correct underlying mutations in stem cells derived from patients, enabling the generation of autologous cell replacement therapy materials that are both genetically corrected and immunologically compatible. This combined approach could significantly enhance the overall efficacy of treatment.

To translate RO therapy into clinical practice, a clear roadmap must be followed. This includes: (1) optimizing GMP-compliant manufacturing to ensure consistent quality, scalability, and tissue maturation through advanced protocols and automated bioreactors; (2) developing robust validation methods to assess organoid functionality and ensure suitability for transplantation; (3) refining surgical techniques to minimize trauma during transplantation; (4) conducting preclinical testing in animal models to evaluate safety, integration, and long-term outcomes; (5) initiating early-phase clinical trials to focus on safety and preliminary efficacy; (6) exploring combined therapeutic approaches, such as integrating gene therapy and gene editing with organoid transplantation; and (7) scaling up manufacturing and deployment for widespread clinical use.

At present, the research community remains focused on addressing challenges related to GMP manufacturing, validation, transplantation, and preclinical testing (Steps 1–4) before advancing to clinical trials and broader implementation (Steps 5–7). However, ongoing research and innovation in these areas will be the key to realizing ROs' full potential in visual rehabilitation for patients with RDD.

##  Financial Support and Sponsorship

NIH R01 EY031834 (MJS); NIH/NEI 1K08EY034912 - 01 (Browne). The authors acknowledge support to the Gavin Herbert Eye Institute at the University of California, Irvine, from an unrestricted grant from Research to Prevent Blindness and from NIH grant P30 EY034070.

##  Conflicts of Interest

None.
